# The effectiveness of pulsed electromagnetic field therapy in patients with shoulder impingement syndrome: A systematic review and meta-analysis of randomized controlled trials

**DOI:** 10.1371/journal.pone.0323837

**Published:** 2025-05-19

**Authors:** Hsun-Yi Wang, Yi-Jen Chen, I-Ching Huang, Chao-Ruei Lin, Ko-Long Lin, Chia-Hsin Chen

**Affiliations:** 1 Department of Physical Medicine and Rehabilitation, Kaohsiung Medical University Hospital, Kaohsiung Medical University, Kaohsiung, Taiwan; 2 Department of Physical Medicine and Rehabilitation, Kaohsiung Municipal Siaogang Hospital, Kaohsiung Medical University, Kaohsiung, Taiwan; 3 Department of Physical Medicine and Rehabilitation, Kaohsiung Medical University Gangshan Hospital, Kaohsiung Medical University, Kaohsiung, Taiwan; 4 Department of Physical Medicine and Rehabilitation, Kaohsiung Medical University Hospital, Kaohsiung, Taiwan; 5 Department of Physical Medicine and Rehabilitation, School of Medicine, College of Medicine, Kaohsiung Medical University, Kaohsiung, Taiwan; 6 Neurotechanology and Assistive Technology Research Center, Kaohsiung Medical University, Kaohsiung, Taiwan; 7 Department of Physical Medicine and Rehabilitation, School of Post-Baccalaureate Medicine, College of Medicine, Kaohsiung Medical University, Kaohsiung, Taiwan; The Chinese University of Hong Kong, HONG KONG

## Abstract

We performed a systematic review and meta-analysis to assess the efficacy of pulsed electromagnetic field (PEMF) therapy in treating patients with shoulder impingement syndrome. We sourced data from PubMed, the Cochrane Library, and Embase databases up until June 19, 2024. Our analysis included randomized controlled trials (RCTs) that evaluated the impact of PEMF therapy on pain levels and functional capacity in these patients. In total, four RCTs involving 252 participants were included. The pooled data indicated that PEMF therapy significantly reduced short-term pain (standardized mean difference [SMD] = -0.34, 95% confidence interval [CI] = -0.66 to -0.01, three RCTs, n = 166) and improved both short-term (SMD = 0.4, 95% CI = 0.08 to 0.73, three RCTs, n = 166) and long-term functional capacity (SMD = 0.6, 95% CI = 0.33 to 0.88, three RCTs, n = 212). The aforementioned results all achieved clinical significance. The observed low heterogeneity for short-term pain, along with short term and long-term functional capacity, highlights the sustained robustness and consistency of the effect on functional capacity over time. These results suggest that PEMF therapy is statistically effective in enhancing short-term pain relief and improving both short-term and long-term functional capacity in patients with shoulder impingement syndrome, with clinically significant benefits. However, the study limitations include a small sample size and variability in PEMF protocols, highlighting the necessity for standardized methodologies in future research.

## Introduction

Shoulder impingement syndrome is a prevalent cause of shoulder pain, accounting for 44% to 65% of all shoulder-related issues.[1,2] This condition is marked by the compression of soft tissues within the subacromial space, primarily impacting the rotator cuff tendons or the bursa.[3] It is a significant source of upper extremity discomfort, leading to functional impairments, restricted shoulder mobility, decreased muscle strength, and a lower quality of life. Non-surgical management options for shoulder impingement syndrome include therapeutic exercises, physical modalities, NSAIDs, and injections.[4–7] Pulsed electromagnetic field (PEMF) therapy, initially developed by Bassett and colleagues in the 1970s, is a non-invasive modality that utilizes pulsed electromagnetic fields to facilitate tissue repair.[8–10] PEMF therapy has demonstrated potential benefits for a range of musculoskeletal conditions, including bone fractures, [11] chronic low back pain, [12] and osteoarthritis [13,14]. Emerging evidence suggests that PEMF therapy may help reduce pain, enhance functional performance, improve quality of life, and boost muscle strength in individuals with shoulder impingement syndrome [15]. PEMF therapy offers distinct advantages over other non-invasive modalities, such as transcutaneous electrical nerve stimulation and ultrasound, by directly modulating cellular processes rather than relying on mechanical or thermal effects. In the context of shoulder impingement syndrome, PEMF therapy may mitigate the catabolic effects of inflammation, particularly those mediated by interleukin-1β, while also promoting tissue repair through the upregulation of vascular endothelial growth factor. These mechanisms make PEMF therapy a promising alternative or adjunct to conventional treatments [16–18]. However, the effectiveness of PEMF therapy in addressing pain and functional outcomes in shoulder impingement syndrome remains debated [15,19]. Prior reviews either lacked sufficient randomized controlled trials or did not specifically evaluate the benefits of PEMF therapy for shoulder impingement syndrome, particularly due to their limited focus on PEMF or inclusion of only a small number of randomized controlled trials [4–6]. Thus, this systematic review and meta-analysis of randomized controlled trials is designed to assess the current evidence on the effectiveness of PEMF therapy, compared to control interventions, in improving pain and functional outcomes in patients with shoulder impingement syndrome.

## Materials and methods

### Study framework, design, and registration

This systematic review and meta-analysis was conducted following the updated Preferred Reporting Items for Systematic Reviews and Meta-Analyses (PRISMA) guidelines [20]. The protocol was registered with PROSPERO, the National Institutes for Health Research’s international prospective register for systematic reviews (registration number: CRD42024532454). The PICO (Patients, Intervention, Comparison, and Outcomes) question posed for this review was: Does PEMF therapy (I) compared to placebo (C) improve pain and functional outcomes (O) in patients diagnosed with shoulder impingement syndrome (P)?

### Data sources and retrieval

We searched the PubMed, Cochrane Library, and Embase electronic databases to identify relevant studies, with no restrictions on language. Pre-determined keywords relating to patient type, intervention, and study design were used. The search terms included combinations such as: (impingement OR rotator cuff OR supraspinatus OR infraspinatus OR subscapularis OR teres minor) for the patient category and “pulsed electromagnetic field (or ‘PEMF’)” for the intervention. Each database was queried using customized search strategies. We identified randomized controlled trials by using the term “random*” or utilizing the specific database filters. Search strategies used in this study are presented in Appendix 1. No language restrictions were applied in our literature search. If any important articles were not retrieved through our search, we manually included them. If data were incomplete, we attempted to contact the authors for unpublished data. Searches were conducted from the databases’ inception to June 19, 2024.

### Eligibility criteria

Two independent reviewers screened the full texts of all retrieved studies to assess their relevance. Only randomized controlled trials investigating PEMF therapy in patients with shoulder impingement syndrome and reporting outcomes like pain or functional capacity were

included. Exclusion criteria applied to studies with patients who had (1) neurological conditions; (2) cervical spine injury; (3) rheumatoid arthritis; or (4) other shoulder pathologies, such as adhesive capsulitis, calcific rotator cuff tendinopathy, partial or full-thickness rotator cuff tears, or trauma. We also excluded non-peer-reviewed articles, conference abstracts, protocol-only studies, and letters to editors. Trials involving combination therapies, where the effects of PEMF could not be separately analyzed, were excluded, but crossover randomized controlled trials were included.

### Data items

Data extraction was performed by two reviewers who independently collected details for both the PEMF and placebo groups. Data items included patient numbers, age, gender, symptom duration, follow-up time, frequency, intensity, and PEMF treatment protocols, as well as baseline treatments. Studies were eligible even if they did not provide all of the aforementioned data. We analyzed outcomes to determine both the short-term effects (post-treatment) and long-term effects (at least 2 months post-treatment) of PEMF therapy. The outcome measurements in this study included pain and functional capacity, with any adverse events also being noted. Missing data and outlier were addressed by contacting authors of the study via email. We also assessed the potential reasons and determined whether the article was included or excluded from this meta-analysis based on their impact on the overall results.

### Risk‐of‐bias assessment

The quality of the studies was assessed independently by two reviewers using the Physiotherapy Evidence Database (PEDro) scale, a validated tool commonly used to evaluate the risk of bias in randomized trials [21]. The PEDro scale includes 11 items, scored as either “Yes” (1 point) or “No” (0 points), depending on whether the criteria were met. One of these items addresses eligibility criteria, which pertains to external validity, and was generally excluded from the total score calculation. The scores from items 2–11 were totaled, with possible scores ranging from 0 to 10. Study quality was rated as poor (<4), fair (4–5), good (6–8), or excellent (9–10) [22]. Any discrepancies between reviewers were resolved through discussion with a third reviewer.

### Statistical analysis

Meta-analysis was conducted using RevMan software (version 5.3; The Cochrane Collaboration, London, UK). To standardize results across different measurement scales, standardized mean differences (SMDs) with 95% confidence intervals (CIs) were used. The data were pooled using a random-effects model due to the variation in methodologies across the included trials. Effect sizes were categorized according to Cohen’s thresholds [23]: trivial (SMD < 0.2), small (SMD 0.2–0.5), moderate (SMD 0.5–0.8), and large (SMD > 0.8) [24,25]. Heterogeneity was assessed using the *I²* test, with ≤50% considered low, 50% < *I²* ≤ 70% indicating moderate, and >70% considered high. Sensitivity analysis was conducted in cases of high heterogeneity to assess the stability of the results. Statistical significance was set at *p* < 0.05 for the z test. For forest plots with more than 10 studies, publication bias was assessed using funnel plots. The certainty in evidence was examined using the Grading of Recommendations, Assessment, Development and Evaluation (GRADE) approach. The certainty of the included randomized controlled trials was determined on the basis of their study design, risk of bias, inconsistency, imprecision, indirectness, publication bias, and effect sizes and their trends. It classifies the quality of evidence on four levels: high, moderate, low, or very low [26].

## Result

### Study collection

**Fig 1:**
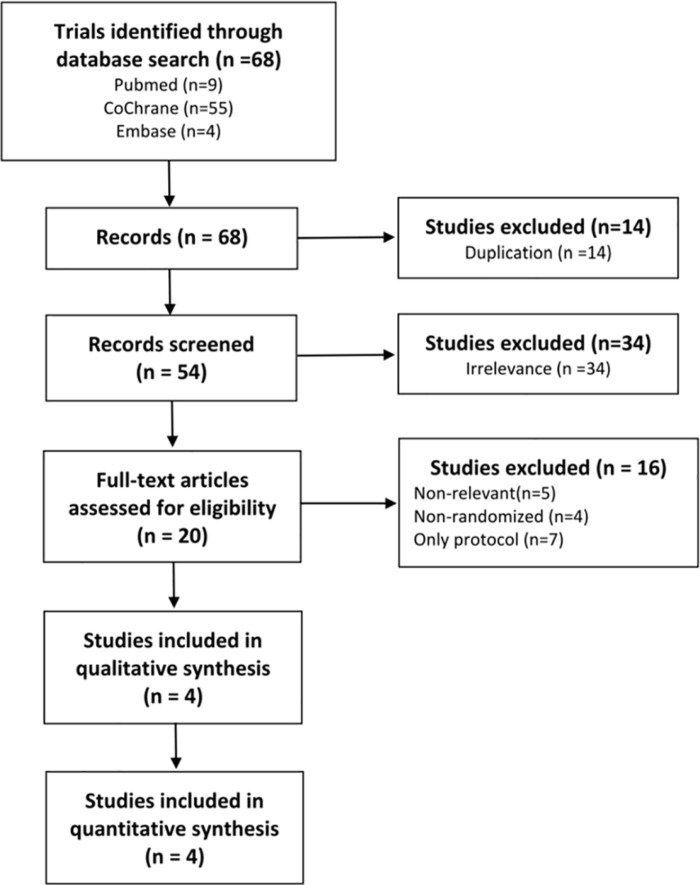
Flowchart of trial selection.

A flowchart of trial selection is presented in Fig 1. Initially, 20 relevant studies were identified. Then, 16 studies were further excluded for the following reasons: being irrelevant to our PICO (n = 5), non-randomized controlled trials (n = 4), and only protocol (n = 7). Finally, four randomized controlled trials involving 252 participants were included in the qualitative and quantitative analysis. All articles were parallel studies [15,19,27,28]. The baseline treatment in three studies was exercise program [15,19,27], and the baseline treatment in one study was focused extracorporeal shock wave therapy.[28] Details about the characteristics of patients and PEMF therapy are shown in Table 1. The duration of the intervention ranges from 3 to 4 weeks, 2–5 times a week. The frequency of PEMF therapy range from 3 Hz to 50 Hz. No challenges or missing data were encountered during the data extraction process.

**Table 1:** Characteristics of the included trials.

Author, year	Group	N	Age in year, mean±SD	Sex, Women/Men	Symptom duration (months, mean±SD)	Intervention (PEMF)			Baseline treatment	Follow-up period	Adherence rate	Outcome measure	Funding sources	Potential conflicts of interest
						Frequency	Intensity	Treatment protocol						
Aktas, 2007 [19]	PEMF	20	48.7 ± 9.0	15/5	4.82 ± 3.75	50 Hz	30G	25 min per session, 5 sessions per week for 3 weeks	Exercise program + cold pack	3 weeks	87%	Pain, function capacity	Not reported	Not reported
	Control	20	53.9 ± 11.2	15/5	4.8 ± 3.47						87%			
de Freitas, 2014 [27]	PEMF	26	50.1 ± 8.2	16/10	N/A	50Hz	20mT or 200G	30 minutes per session, 3 sessions per week for 3 weeks	Exercise program	3, 9 and 12 weeks	100%	Pain, function capacity	None	Not reported
	Control	30	50.8 ± 9.6	20/10	N/A						100%			
Kandemir, 2023 [15]	PEMF	40	49.82 ± 8.05	25/15	22 ± 17.7	50 Hz	85G	30 minutes per day, 5 days per week for 4 weeks	Exercise program	4 and 12 weeks	100%	Pain, function capacity	None	None
	Control	40	49.62 ± 9.40	24/16	21.2 ± 19						100%			
Klüter, 2018 [28]	PEMF	44	50.21 ± 8.5	23/21	8.42 ± 7.96	3 Hz	80mT	20 minutes per session, two sessions per week for 4 weeks	Focused ESWT	6, 12 and 24 weeks	100%	Pain, function capacity	Not reported	None
	Control	42	49.21 ± 7.3	22/20	8.12 ± 7.34						100%			

N, number of patients; SD, standard difference; PEMF, pulsed electromagnetic fields therapy; mT, milli-Tesla; G, Gauss; ESWT, extracorporeal shock wave therapy; N/A,

*Petechiae, small subcutaneous hematomas, or erythema.

### Risk‐of‐bias assessment

The quality of the randomized controlled trials included in this review was independently evaluated by two reviewers using the PEDro scale. The PEDro scores for all studies ranged from 8 to 9. According to these scores, two studies were classified as “good” [19,27], while the remaining two were categorized as “excellent” [15,28]. Detailed information on the risk of bias assessment can be found in Table 2.

**Table 2:** Summary of methodological quality based on PEDro scale.

Study	1*	2	3	4	5	6	7	8	9	10	11	Total
Aktas, 2007 [19]		∨		∨	∨	∨	∨	∨		∨	∨	**8**
de Freitas, 2014 [27]	∨	∨	∨	∨	∨	∨	∨			∨	∨	**8**
Klüter, 2018 [28]		∨	∨	∨	∨		∨	∨	∨	∨	∨	**9**
Kandemir, 2023 [15]	∨	∨	∨	∨	∨	∨	∨	∨		∨	∨	**9**

1, eligibility criteria and source of participants; 2, random allocation; 3, concealed allocation; 4, baseline comparability; 5, blinded participants; 6, blinded therapists; 7, blind assessors; 8, adequate follow-up; 9, intention-to-treat analysis; 10, between-group comparisons; 11, point estimates and variability. *Not included in the calculation of the total score.

### Synthesis of results

In total, four randomized controlled trials were included in the meta-analysis.

### Pain scores

**Fig 2:**
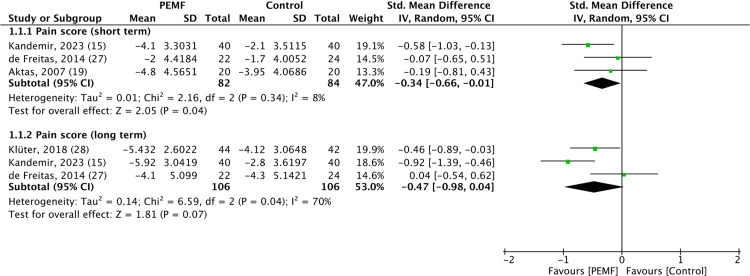
Forest plot of the effect of pulsed electromagnetic fields therapy on pain scores, showing that pulsed electromagnetic fields therapy resulted in significantly better short-term pain scores than placebo. Analysis was performed using standard mean difference with a random effects model, and heterogeneity was assessed by the *I²* statistic.

Pain scores, assessed using a visual analog scale, were reported in all included studies. These scores were evaluated at two different time points: immediately following the treatment (short-term) and at follow-up (at least 2 months after treatment, long-term). Our analysis indicated that PEMF therapy statistically significantly alleviated short-term pain compared to control groups, with short-term pain scores achieving the minimal clinically important difference. Additionally, three randomized controlled trials were analyzed, revealing minimal heterogeneity among the studies (SMD = −0.34, 95% CI −0.66 to −0.01, *p* value = 0.04, *I²* = 8%) [15,19,27]. In contrast, for long-term pain, three studies were reviewed, but no statistically significant difference was found (SMD= −0.47, 95% CI −0.98 to 0.04, *p* value = 0.07) [15,27,28]. The forest plot illustrating the pain scores is shown in Fig 2.

### Functional capacity

**Fig 3:**
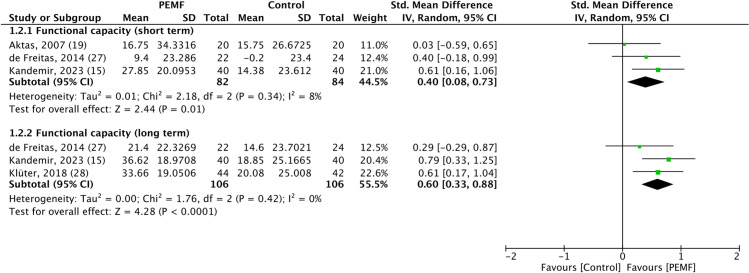
Forest plot of the effect of pulsed electromagnetic fields therapy on functional capacity, revealing that pulsed electromagnetic fields therapy resulted in significantly better short-term and long-term functional capacity than placebo. Analysis was performed using standard mean difference with a random effects model, and heterogeneity was assessed by the *I²* statistic.

Functional capacity, measured using the Constant Murley Score (CMS), was reported in all studies included in the review. This measure was assessed at two distinct time points: immediately following the treatment (short-term) and at follow-up (at least 2 months after treatment, long-term). Our analysis revealed that PEMF therapy statistically significantly enhanced both short-term and long-term functional capacity compared to control groups, with these improvements reaching clinically meaningful thresholds. For short-term functional capacity, three randomized controlled trials were evaluated, demonstrating minimal heterogeneity (SMD = 0.4, 95% CI 0.08 to 0.73, *p* value = 0.01, *I*^*2*^ = 8%) [15,19,27]. Similarly, for long-term functional capacity, three randomized controlled trials showed significant improvements with low heterogeneity (SMD = 0.6, 95% CI 0.33 to 0.88, *p* value<0.0001, *I*^*2*^ = 0%) [15,27,28]. The forest plot depicting functional capacity outcomes is shown in Fig 3.

The Grading of Recommendations, Assessment, Development and Evaluation (GRADE) methodology indicated a high quality of evidence for both short-term pain scores and functional capacity, evaluated at both short-term and long-term intervals. In contrast, the assessment revealed a moderate quality of evidence for long-term pain scores, attributed to observed inconsistencies in the data [15,27,28]. Table 3 presents the evidence proﬁle of it.

**Table 3:** Grading of recommendations, assessment, development and evaluation (GRADE) of studies with outcomes.

Question: Effects of pulsed electromagnetic field therapy compared with placebo on patients with shoulder impingement syndrome												
Certainty assessment							№ of patients		Effect		Certainty	Importance
№ of studies	Study design	Risk of bias	Inconsistency	Indirectness	Imprecision	Other considerations	PEMF	Control	Relative (95% CI)	Absolute (95% CI)		
Pain scores (short term)												
3	randomised trials	not serious	not serious	not serious	not serious	none	82	84	–	SMD 0.34 lower (0.66 lower to 0.01 lower)	⊕⊕⊕⊕High	IMPORTANT
Pain scores (long term)												
3	randomised trials	not serious	serious^a^	not serious	not serious	none	106	106	–	SMD 0.47 lower (0.98 lower to 0.04 higher)	⊕⊕⊕○ Moderate	IMPORTANT
Functional capacity (short term)												
3	randomised trials	not serious	not serious	not serious	not serious	none	82	84	–	SMD 0.4 higher (0.08 higher to 0.73 higher)	⊕⊕⊕⊕High	IMPORTANT
Functional capacity (long term)												
3	randomised trials	not serious	not serious	not serious	not serious	none	106	106	–	SMD 0.6 higher (0.33 higher to 0.88 higher)	⊕⊕⊕⊕High	IMPORTANT

^a^The heterogeneity is high (*I*^*2*^ = 70%)

**Fig 4:**
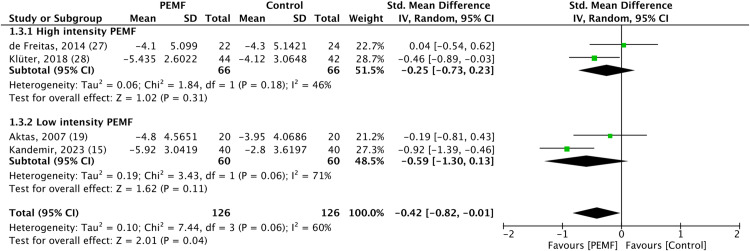
Forest plot of subgroup analysis comparing high- and low-intensity pulsed electromagnetic field therapy effects on pain scores, showing that no significant differences were noted in pain scores for high- and low-intensity pulsed electromagnetic field therapy when each was compared to placebo. Analysis was performed using standard mean difference with a random effects model, and heterogeneity was assessed by the *I²* statistic.

**Fig 5:**
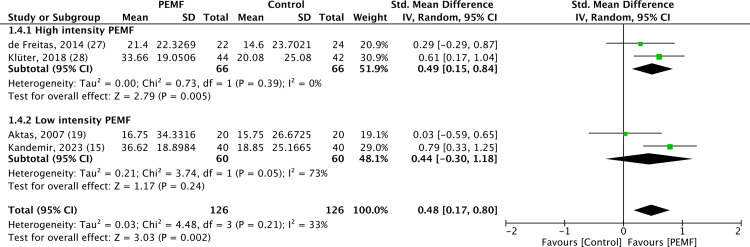
Forest plot of subgroup analysis comparing high- and low-intensity pulsed electromagnetic field therapy effects on functional capacity, revealing that high-intensity pulsed electromagnetic fields therapy resulted in significantly functional capacity than placebo. Analysis was performed using standard mean difference with a random effects model, and heterogeneity was assessed by the *I²* statistic.

In the subgroup analysis of PEMF intensity, high-intensity PEMF therapy (>10 millitesla) was associated with a statistically significant improvement in functional capacity, while no significant difference was observed for pain scores. Conversely, low-intensity PEMF therapy (<10 millitesla) showed no statistically significant difference in either pain scores or functional capacity. The forest plots depicting subgroup analysis for pain scores and functional capacity was shown in Figs 4 and 5.

## Discussion

Our study demonstrated that PEMF therapy for patients with shoulder impingement syndrome led to significantly greater improvements in short-term pain scores and in both short-term and long-term functional capacity compared to control groups. Previous research has established that a SMD of 0.2 is a threshold for distinguishing between small and moderate effects and for clinical significance [25,29,30]. In our study, the SMD values for short-term pain, short-term functional capacity, and long-term functional capacity were 0.34, 0.4, and 0.6, respectively, indicating clinical significance.

Three systematic reviews and meta-analyses have addressed conservative treatments for shoulder impingement syndrome, although with varying methodologies and conclusions regarding PEMF therapy [4–6]. The study by Gebremariam et al. conducted a systematic review of physiotherapy for shoulder impingement syndrome but did not perform a meta-analysis [6]. Dong et al. performed a network meta-analysis that included only two randomized controlled trials on PEMF, comparing it with other treatments, and found no additional benefit of combining PEMF therapy with exercise for pain relief and functional improvement [5]. The meta-analysis by Steuri et al. evaluated conservative treatments for shoulder impingement syndrome, but PEMF therapy was not analyzed independently and was grouped with other modalities such as ultrasound and transcutaneous electrical nerve stimulation under the category of miscellaneous treatments [4]. These studies highlighted the limited evidence supporting the use of PEMF therapy. To our knowledge, this is the first meta-analysis specifically evaluating PEMF therapy’s effectiveness for shoulder impingement syndrome. Our study also examines both short-term and long-term benefits of PEMF therapy, identifying four randomized controlled trials that compared PEMF therapy to control groups, including two trials not covered in previous meta-analyses. Therefore, our study contributes additional evidence supporting the effectiveness of PEMF therapy in improving pain scores and functional capacity in these patients.

Understanding why PEMF therapy benefits patients with shoulder impingement syndrome is crucial. This condition involves pain from inflammation and damage to structures in the subacromial space. PEMF therapy is thought to facilitate calcium ion migration into cells, enhance calcium ion binding to calmodulin (a protein that aids nitric oxide release and growth factor secretion), and modulate immune cell function by lowering interleukin levels and suppressing the catabolic effects of interleukin-1β-induced pro-inflammatory signals, which promote tendinopathy by activating matrix metalloproteinases and leading to extracellular matrix degradation. Additionally, PEMF therapy supports the conversion of monocytes to macrophages, helping remove microorganisms, foreign substances, and dead cells from the affected area [31–33]. Moreover, PEMF exposure has been demonstrated to upregulate collagen type I expression and elevate the levels of interleukin-10 and vascular endothelial growth factor, both of which play key roles in promoting tendon healing [16–18]. We hypothesize that PEMF therapy alleviates pain and enhances functional capacity by reducing inflammation and promoting tissue regeneration.

Currently, there is no universally accepted cut-off for frequency or intensity in PEMF therapy, with optimal parameters varying by clinical application and patient condition. The literature suggests that low-frequency ranges typically fall between 5 and 50 Hz [34,35]. while low-intensity PEMF therapy is defined as below 10 millitesla [36]. According to the literature, a 50 Hz threshold is frequently used to distinguish PEMF therapy frequencies. As all studies included in our analysis utilized frequencies within this threshold, aligning with standard cut-off values, we did not perform a frequency-based subgroup analysis. Some trials indicate that PEMF therapy may effectively reduce pain and inflammation [35,37]. However, low-intensity PEMF therapy lacks the dynamic oscillations necessary to activate signaling pathways for tissue regeneration, potentially leading to suboptimal healing outcomes in conditions like tendinopathy and delayed bone healing [38–40]. Our study found that high-intensity PEMF therapy improved functional capacity without significantly affecting pain levels, whereas low-intensity PEMF therapy showed no significant improvements in either measure, indicating the advantages of high-intensity PEMF therapy for shoulder impingement syndrome. Nevertheless, our study included only two randomized controlled trials for both high-intensity and low-intensity PEMF groups, respectively. Therefore, additional randomized controlled trials are required in the future.

Among the four randomized controlled trials included in our analysis, one employed extracorporeal shock wave therapy as the baseline treatment [28], whereas the others used exercise (with or without cold packs) as their baseline intervention [15,19,27]. As the study by Klüter et al. was included only in the assessment of long-term outcomes, we conducted a sensitivity analysis to determine whether the use of extracorporeal shock wave therapy as a baseline treatment may have influenced our findings regarding long-term outcomes. Excluding this study, the long-term pain scores remained non-significant (SMD= −0.46, 95% CI −1.4 to 0.48, *p* value = 0.34), while the long-term functional capacity continued to demonstrate significant improvement, with no notable change in effect size (SMD = 0.57, 95% CI 0.09 to 1.06, *p* value = 0.02, *I*^*2*^ = 43%). Thus, in sensitivity analysis, no obvious changes were found for the effect of PEMF therapy on the long-term outcomes in shoulder impingement syndrome.

Adverse events, such as petechiae, small subcutaneous hematomas, or erythema, were reported in both the treatment and control groups, but these resolved within 24 hours and no severe events were noted [28]. However, due to inconsistent reporting of adverse events in the included trials, we cannot definitively assess the safety of PEMF therapy for shoulder impingement syndrome. Future studies should emphasize standardized and comprehensive adverse event reporting to enable more accurate assessments of treatment safety.

This meta-analysis has several strengths. Firstly, this study is the first meta-analysis of randomized controlled trials focusing on the efficacy of PEMF therapy for patients with shoulder impingement syndrome. Secondly, our study distinguishes the benefits of PEMF therapy in both short-term and long-term contexts. Thirdly, the quality of all the selected randomized controlled trials was at least good, as indicated by PEDro scores [15,19,27,28]. Notably, most of the included studies featured blinding of participants, therapists, and assessors, with the exception of Klüter et al. [28], which did not involve therapist blinding. Lastly, no signiﬁcant inconsistency was noted in our results, which refers to good coherence.

This study also has several limitations. Firstly, the selected randomized controlled trials employed diverse PEMF therapy protocols, varying in frequency, intensity, duration per session, total number of sessions, and overall treatment duration. Secondly, one trial included in our meta-analysis utilized extracorporeal shock wave therapy, which may have influenced the outcomes. Thirdly, our meta-analysis included only four randomized controlled trials, all of which exhibited geographic concentration. This limitation may have hindered our ability to detect subtle differences in treatment effects and restricted the generalizability of the findings. Forth, the maximum follow-up duration in the included studies was limited to 24 weeks. Fifth, the studies included in our analysis demonstrated variability in the reporting of adverse events and a lack of patient-reported outcome measures. Future research should focus on large-scale randomized controlled trials with standardized PEMF protocols, and include studies assessing long-term safety and efficacy to strengthen the evidence base.

## Conclusion

This meta-analysis indicates that PEMF therapy significantly improve short-term pain scores and both short-term and long-term functional capacity in patients with shoulder impingement syndrome, and may complement existing conservative approaches in its management. However, several limitations were identified in our analysis, and further studies are needed to validate these findings and refine treatment protocols.

## Supporting information

S1 File Search strategy. (DOCX)

S2 File PRISMA checklist. (DOCX)
