# Supplementary material for: The effectiveness of pulsed electromagnetic field therapy in patients with shoulder impingement syndrome: A systematic review and meta-analysis of randomized controlled trials
Source: PLoS One. 2025 May 19;20(5):e0323837. doi: 10.1371/journal.pone.0323837 (PMC12088032; doi:10.1371/journal.pone.0323837)
Supplement: S1 File — (DOCX) [file pone.0323837.s001.docx]

**Appendix 1. Search strategy**

**Keywords for search of different electronic databases**

| Data base | Search terms for query |
| --- | --- |
| PubMed |  |
| #1 | impingement |
| #2 | rotator cuff |
| #3 | supraspinatus |
| #4 | infraspinatus |
| #5 | subscapularis |
| #6 | teres minor |
| #7 | #1 OR #2 OR#3 OR #4 OR #5 OR #6 |
| #8 | pulsed electromagnetic field |
| #9 | PEMF |
| #10 | #8 OR #9 |
| #11 | #7 AND #10 |
| Filters: | Pubmed: Randomized Controlled Trial |

| Data base | Search terms for query |
| --- | --- |
| Embase |  |
| #1 | impingement |
| #2 | rotator cuff |
| #3 | supraspinatus |
| #4 | infraspinatus |
| #5 | subscapularis |
| #6 | teres minor |
| #7 | #1 OR #2 OR#3 OR #4 OR #5 OR #6 |
| #8 | pulsed electromagnetic field |
| #9 | PEMF |
| #10 | #8 OR #9 |
| #11 | #7 AND #10 |
| Filters: | Pubmed: Randomized Controlled Trial |

| Data base | Search terms for query |
| --- | --- |
| Cochrane |  |
| #1 | impingement |
| #2 | rotator cuff |
| #3 | supraspinatus |
| #4 | infraspinatus |
| #5 | subscapularis |
| #6 | teres minor |
| #7 | #1 OR #2 OR#3 OR #4 OR #5 OR #6 |
| #8 | pulsed electromagnetic field |
| #9 | PEMF |
| #10 | #8 OR #9 |
| #11 | #7 AND #10 |
| Filters: | Pubmed: Trial |
